# Nationwide implementation of lenalidomide maintenance in multiple myeloma: A retrospective, real‐world study

**DOI:** 10.1002/jha2.881

**Published:** 2024-03-27

**Authors:** Mads Harsløf, Iman Chanchiri, Trine Silkjær, Ulf Christian Frølund, Elena Manuela Teodorescu, Kristina Buchardi Nielsen, Per Ishøy Nielsen, Per Trøllund Pedersen, Katrine Fladeland Iversen, Thomas Lund, Kirsten Grønbæk, Sigrun Thorsteinsdottir, Annette Vangsted, Agoston Gyula Szabo

**Affiliations:** ^1^ Department of Hematology Rigshospitalet Copenhagen Denmark; ^2^ Biotech Research and Innovation Centre University of Copenhagen Kobenhavn Denmark; ^3^ Department of Hematology Odense University Hospital Odense Denmark; ^4^ Department of Hematology Aarhus University Hospital Aarhus Denmark; ^5^ Department of Hematology Zealand University Hospital Roskilde Denmark; ^6^ Department of Hematology Aalborg University Hospital Aalborg Denmark; ^7^ Department of Hematology Regionshospitalet Gødstrup Herning Denmark; ^8^ Department of Hematology Esbjerg Hospital Esbjerg Denmark; ^9^ Department of Hematology Vejle Hospital Vejle Denmark

**Keywords:** lenalidomide maintenance, multiple myeloma, real‐world evidence, transplant eligible

## Abstract

Lenalidomide maintenance (LM) has shown benefit in progression‐free survival (PFS) and overall survival (OS) in clinical trials. LM is the recommended standard of care in patients with newly diagnosed multiple myeloma (MM) after high‐dose melphalan and autologous stem cell transplantation (HDM‐ASCT). In Denmark, LM has been approved and publicly funded for all patients treated with HDM‐ASCT since June 2019. Patients with newly diagnosed MM treated with their first HDM‐ASCT between June 2019 and March 2022 were included and followed until data cut‐off in June 2023. To compare outcomes, a historical pre‐LM cohort from the Danish MM Registry, consisting of 364 MM patients treated with HDM‐ASCT between June 2015 and June 2019, was used. Among 364 patients treated with HDM‐ASCT after June 2019, 22.3% received consolidation therapy and 3.7% underwent tandem HDM‐ASCT. During follow‐up, 297 patients (81.6%) initiated maintenance therapy, with 277 (76.1%) receiving LM. Overall, 145 patients (52.3%) discontinued LM most commonly due to toxicity 75 (51.7%), with fatigue (30.7%), cytopenia (25.3%), and neuropathy (17.3%) being the main reasons. In a 6‐month landmark analysis, early discontinuation did not negatively impact PFS or OS. The LM cohort had similar PFS, and OS compared to the pre‐LM cohort. The 3‐year PFS and OS rates in the LM cohort were 61% and 86%, respectively, while the pre‐LM cohort had a 3‐year PFS of 55% and a 3‐year OS of 89%. In conclusion, the introduction of LM as a nationwide treatment option in Denmark did not lead to improved clinical outcomes.

## 1 INTRODUCTION

Lenalidomide maintenance (LM) has shown progression‐free survival (PFS) and overall survival (OS) benefit in clinical trials and is the recommended standard of care in patients with newly diagnosed multiple myeloma (MM) after high‐dose melphalan and autologous stem cell transplantation (HDM‐ASCT) [1, 2, 3, 4, 5]. Although LM was generally well tolerated in randomized controlled trials (RCTs), serious adverse events were uniformly more common in the LM group compared to observation [1, 2, 3, 4]. Especially worrisome was the increased incidence of second primary malignancies following LM [6]. Although RCTs are needed to evaluate efficacy and ascertain causality, their findings do not always seamlessly translate into real‐world clinical practice [7]. Conversely, real‐world data are limited by the non‐random assignment of treatments and difficulties in establishing an appropriate timepoint for starting follow‐up in survival analysis [8]. The latter is especially challenging as LM is started several months after HDM‐ASCT and may be delayed by consolidation therapy or treatment complications. A failure to account for this introduces a guarantee‐time bias, which may inflate the estimated benefit of LM in survival analysis. Other real‐world studies found that LM was associated with better PFS and OS [9, 10, 11]. Another found that LM was only associated with PFS benefit but not OS benefit [12].

In Denmark, LM until progression or toxicity has been approved and is publicly funded for all newly diagnosed MM patients treated with HDM‐ASCT since June 2019. The fact that LM was made available nationwide simultaneous across all regions creates a unique opportunity to study the real‐world use pattern, efficacy, and adverse effects of LM in an unselected, population‐based cohort without referral bias.

## 2 METHODS

### 2.1 The LM cohort

Patients with newly diagnosed MM fulfilling the CRAB(hypercalcemia, renal failure, anemia or osteolytic bone lesions) or SLiM‐CRAB criteria according to the definitions of the International Myeloma Working Group (IMWG) [13] treated with their first HDM‐ASCT between June 1, 2019 and March 1, 2022 were included and followed until data cut‐off on June 14, 2023. Patients were identified from the local transplantation registers.

Medical doctors at eight respective departments of hematology reviewed the electronic records of all patients retrospectively. For each patient receiving maintenance therapy, the used drugs, the dosage, the date of initiation, the date of discontinuation, and the cause of discontinuation were recorded. Toxicities were registered only if they were the cause of dose reduction or discontinuation of LM. Non‐melanoma skin cancers were not reported, but all other second primary malignancies were reported according to the International Classification of Diseases 10th revision (ICD‐10). Lines of therapy and responses were evaluated according to the IMWG recommendations [14].

### 2.2 The pre‐LM cohort

A historical pre‐LM cohort consisting of MM patients treated with their first HDM‐ASCT between June 1, 2015 and June 1, 2019 was used for comparison. Information on vital status and emigration until November 1, 2022 was retrieved from the Danish Civil Registration System. Information on progression was retrieved from the Danish MM Registry. Due to the delay in this registry compared with the Danish Civil Registration System, the follow‐up for progressive disease ended on December 31, 2020.

### 2.3 Baseline characteristics

Baseline clinical characteristics, radiological findings, laboratory measurement, and data on cytogenetics were acquired from the Danish MM Registry. This database has previously been validated and contains clinical data for every patient diagnosed with MM since 2005 [15]. CRAB criteria were measured at the time of diagnosis and defined as a calcium level above 1.35 mmol/L, hemoglobin below 10 g/dL (6.2 mmol/L), osteolytic bone lesions on imaging, or creatinine above 177 µmol/L. Revised international staging system (ISS) score was calculated according to IMWG criteria [16]. For lactate dehydrogenase, a cut‐off of 205 IU/L was used according to Danish guidelines [16]. High‐risk cytogenetics were defined as the presence of either *t*(4;14), *t*(14;16), or del17p in diagnostic fluorescence in situ hybridization analysis. Standard‐risk cytogenetics were defined as the lack of high‐risk cytogenetics.

### 2.4 Statistical analysis

We presented number and percentage for categorical variables, and median and interquartile range for continuous variables. Median follow‐up and median duration of maintenance therapy were calculated using the reverse Kaplan–Meier estimator method. Causes of lenalidomide discontinuation were visualized using non‐parametric Aalen–Johansen estimates of the cumulative incidence function with progression, toxicity, and other reasons treated as competing events. To avoid the risk of guarantee‐time bias, several strategies were applied [17]. Early discontinuation of LM was studied using landmark analysis at 6 months after initiation of LM. Patients with death or progression before this date were excluded for analysis, and patients were grouped according to whether they had discontinued LM at the landmark date. Sensitivity analysis was conducted using a 12‐month landmark. For comparison with the pre‐LM cohort, survival time was calculated from date of ASCT to progression or death for PFS, and death of any cause for OS. This mimics an intention to treat analysis as not all patients in the LM cohort were treated with LM. For all survival analyses, unadjusted Kaplan–Meier curves are shown. Cox proportional hazards regression was used to calculate hazard ratios (HR). Multivariate adjustment was made for age and sex as well as known risk factors for worse outcome: ISS and high‐risk cytogenetics. Pearsons's chi‐squared test was used to compare proportion with very good partial response (VGPR) or better to subsequent line of therapy. Statistical analyses were performed using R statistical software version 4.2.2 (2022‐10‐31).

## 3 RESULTS

We identified 364 patients treated with HDM‐ASCT after the introduction of LM. Median follow‐up from HDM‐ASCT was 31 months, median age was 61.1 years, 56.6% were male, 31% had anemia, 10.5% had renal failure, and 19.8% had hypercalcemia at diagnosis. Cytogenetic data were available in 79.1% of patients, and 25.0% of these had high‐risk cytogenetics defined as the presence of *t*(4;14), *t*(14;16), or del17p (Table 1).

**TABLE 1:** Baseline data of 728 patients treated with high‐dose melphalan and autologous stem cell transplantation (HDM‐ASCT) in Denmark before and after the approval of lenalidomide maintenance (LM) in June 2019.

	Pre‐LM (*N* = 364)	LM (*N* = 364)	Overall (*N* = 728)
Age	61.8 [55.1, 66.5]	61.1 [55.3, 66.1]	61.3 [55.3, 66.3]
Sex			
Female	146 (40.1%)	158 (43.4%)	304 (41.8%)
Male	218 (59.9%)	206 (56.6%)	424 (58.2%)
WHO performance status			
0–1	309 (86.8%)	286 (86.1%)	595 (86.5%)
>1	47 (13.2%)	46 (13.9%)	93 (13.5%)
Bone marrow infiltration (%)	40.0 [20.0, 60.0]	50.0 [20.0, 70.0]	45.0 [20.0, 70.0]
Myeloma protein			
IgA	83 (23.2%)	58 (17.8%)	141 (20.6%)
IgG	198 (55.5%)	189 (58.0%)	387 (56.7%)
Light chain	56 (15.7%)	65 (19.9%)	121 (17.7%)
Other	20 (5.6%)	14 (4.3%)	34 (5.0%)
Calcium ion > 1.35 (mmol/L)	71 (24.1%)	58 (19.8%)	129 (21.9%)
Creatinine > 177 (µmol/L)	49 (13.5%)	35 (10.5%)	84 (12.1%)
Hemoglobin < 6.2 (mmol/L)	133 (36.6%)	103 (31.0%)	236 (34.0%)
Osteolytic lesions	291 (80.6%)	250 (75.8%)	541 (78.3%)
Amyloidosis	4 (1.1%)	3 (0.9%)	7 (1.0%)
Dialysis	9 (2.5%)	6 (1.8%)	15 (2.2%)
Spinal cord compression	25 (6.9%)	26 (7.9%)	51 (7.4%)
Beta‐2‐mikroglobulin (mg/L)	3.85 [2.57, 6.22]	3.60 [2.40, 5.70]	3.71 [2.50, 6.00]
Albumin (g/L)	35.0 [30.3, 39.0]	36.0 [30.5, 40.0]	36.0 [30.0, 40.0]
LDH > 205 (IU/L)	112 (30.9%)	112 (34.3%)	224 (32.5%)
ISS			
I	103 (30.5%)	101 (32.3%)	204 (31.3%)
II	135 (39.9%)	124 (39.6%)	259 (39.8%)
III	100 (29.6%)	88 (28.1%)	188 (28.9%)
R‐ISS			
I	57 (17.6%)	48 (17.3%)	105 (17.4%)
II	206 (63.6%)	175 (62.9%)	381 (63.3%)
III	61 (18.8%)	55 (19.8%)	116 (19.3%)
Cytogenetics available	348 (95.6%)	288 (79.1%)	632 (86.8%)
Cytogenetic alteration			
*t*(4,14)	33 (9.5%)	33 (11.5%)	66 (10.4%)
*t*(14,16)	11 (3.2%)	11 (3.8%)	22 (3.5%)
del17p	26 (7.5%)	38 (13.2%)	64 (10.1%)
High‐risk FISH^a^	66 (19.0%)	72 (25.0%)	138 (21.7%)
Induction regimen			
VRD	31 (8.5%)	234 (64.3%)	265 (36.4%)
VCD	294 (80.8%)	100 (27.5%)	394 (54.1%)
Other	39 (10.7%)	30 (8.2%)	69 (9.5%)
VGPR or better	284 (78.7%)	308 (84.8%)	592 (81.8%)
Days from diagnosis to HDM‐ASCT	146 [130, 168]	154 [139, 181]	149 [135, 175]

*Note*: For continuous variables, median and interquartile range (IQR) are reported, and for categorical variables, number of observations and percentage are shown.

Abbreviations: FISH, fluorescence in situ hybridization; HDM‐ASCT, high‐dose melphalan and autologous stem cell transplantation; IgA, immunoglobulin A; IgG, immunoglobulin G; ISS, International Staging System; LDH, lactate dehydrogenase; R‐ISS, revised‐ISS; VCD, bortezomib–cyclophospamide–dexamethason; VGPR, very good partial response; VRD, bortezomib–lenalidomide–dexamethason.

^a^ High‐risk cytogenetic abnormalities were defined as *t*(4;14), *t*(14;16), and del(17p).

Bortezomib–lenalidomide–dexamethasone (VRD) or bortezomib–cyclophosphamide–dexamethasone (VCD) were used as induction in 64.3% and 27.5% of patients, respectively. After HDM‐ASCT, the VGPR or better rate was 84.8%. Consolidation therapy was used in 22.3%, and tandem HDM‐ASCT in 3.7% of patients.

### 3.1 Lenalidomide maintenance

**FIGURE 1:**
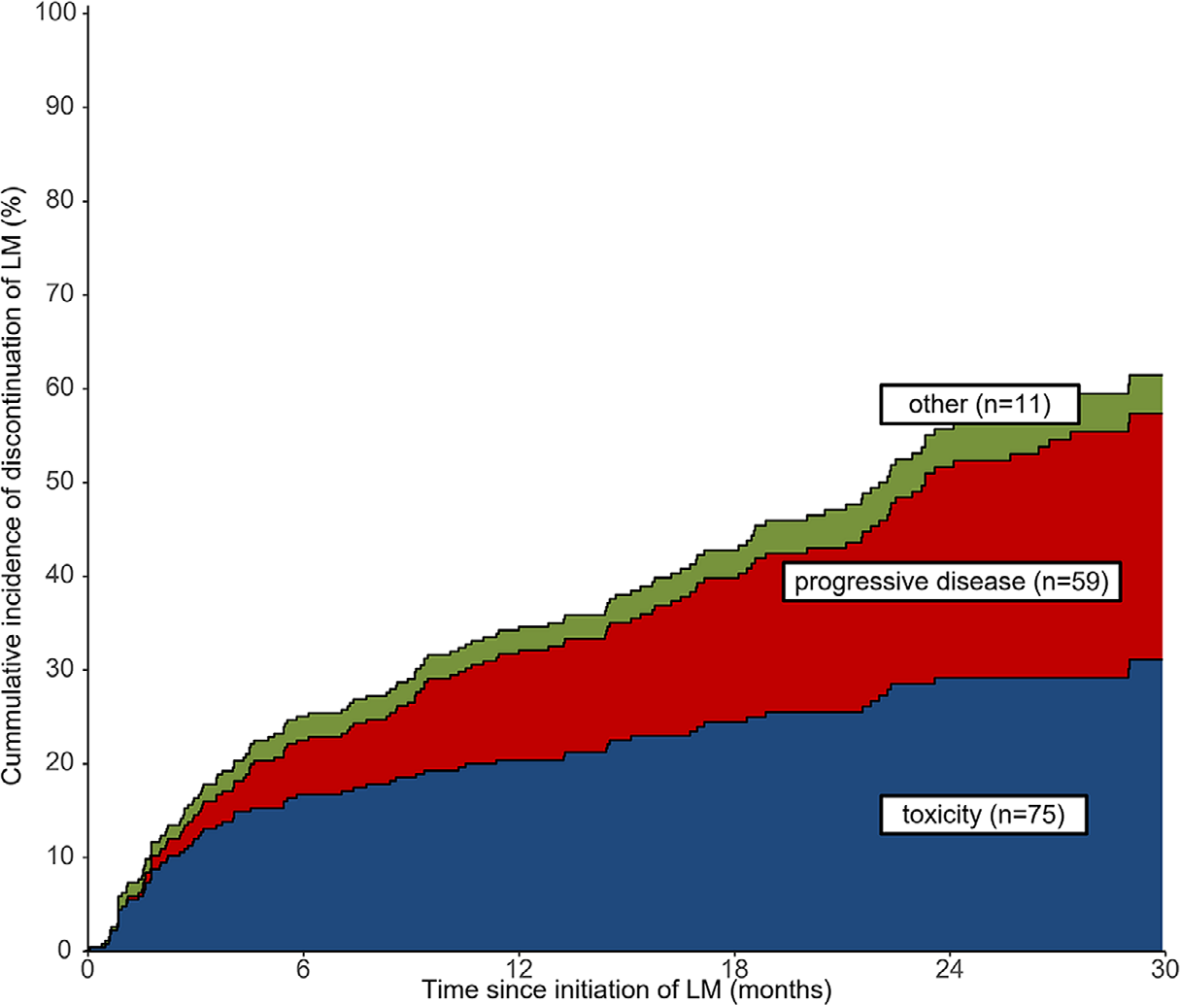
The cumulative incidence and causes of discontinuation of lenalidomide maintenance (LM). Analysis was conducted in 277 patients receiving LM and shows stacked cumulative incidence curves for competing risks of lenalidomide discontinuation.

In 297 (81.6%) patients, maintenance therapy was initiated during follow‐up, of which 277 (76.1%) patients were treated with LM, and 20 (5.5%) patients received other maintenance, primarily daratumumab (*n* = 10) or ixazomib (*n* = 9). From HDM‐ASCT, the median time to initiation of LM was 107 days for those not receiving consolidation therapy. The most frequently used dosing was 10 mg (93.3%) for 21 days (82.3%). Lenalidomide was dose reduced in 37.5% of patients; the main reasons were cytopenia (49.0%), fatigue (23.1%), and rash (12.5%) (Table S2). Median time on LM was 22.6 months. The estimated 12‐month discontinuation rate was 34.5% (Figure 1), and a total of 145 (52.3%) patients discontinued LM; most frequently due to toxicity (51.7%) and progressive disease (40.7%). The main toxicities leading to discontinuation were fatigue (30.7%), cytopenia (25.3%), and neuropathy (17.3%) (Table S3). Early discontinuations within the first 6 months of LM were primarily caused by toxicity. At later time points, progression was a more dominant cause of discontinuation (Figure 1).

### 3.2 Early discontinuation

**FIGURE 2:**
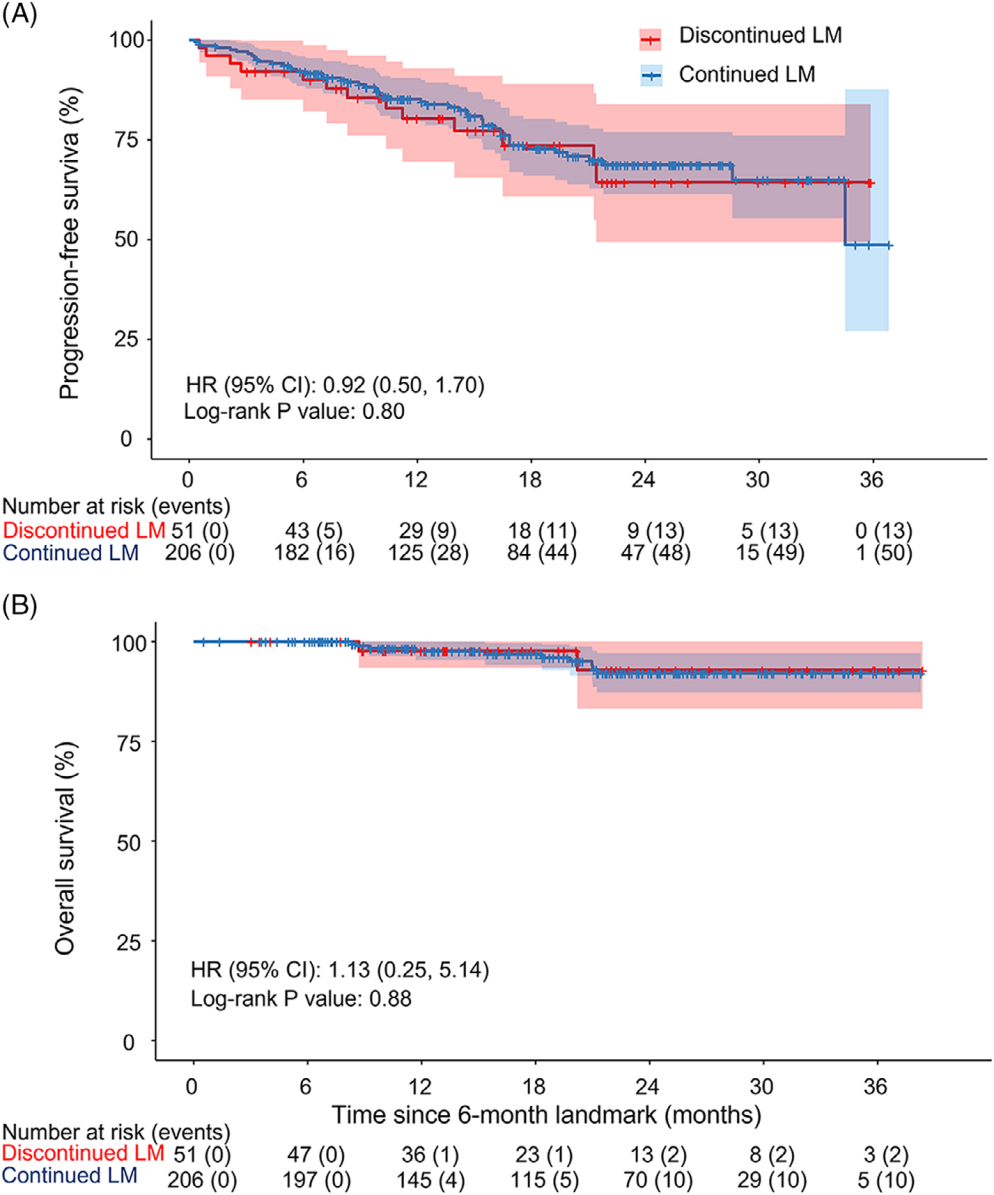
Progression‐free survival (A) and overall survival (B), landmark analysis 6 months after initiation of lenalidomide maintenance (LM). Kaplan–Meier curves for landmark analysis 6 months post initiation of LM. Eighteen progressed before landmark date, one died, and one had insufficient follow‐up time. Unadjusted hazard ratios (HR) were calculated using cox proportional hazards regression. CI, confidence interval.

During follow‐up, 119 (32.7%) patients had an event of progressive disease or death. To assess whether early discontinuation increased the risk of progression, we did a 6‐month landmark analysis. At 6 months after initiation of lenalidomide, patients without progression or death were grouped into those still on LM (*n* = 206) and those who had stopped LM (*n* = 51). Most baseline characteristics were similar in the two groups, although there was a higher prevalence of renal insufficiency, del17p and better WHO performance status in patients who had discontinued lenalidomide (Table S1). No difference in PFS or OS was observed (Figure 2). As a sensitivity analysis, a 12‐month landmark analysis was done with similar result (Figure S1).

### 3.3 Subsequent line of therapy

During follow‐up, 108 patients received a subsequent line of therapy. Of these, 76 (70.4%) received a daratumumab‐containing regimen. The most frequently used combination in patients not treated with LM was daratumumab–lenalidomide–dexamethasone (DRD, 50.0 %), while it was DRD (26.3%) and daratumumab–bortezomib–dexamethasone (22.4%) in patients treated with LM (Table S4). The rate of VGPR or better achieved with the subsequent line of therapy was 45.5% in patients who had received LM versus 53.8% in patients who had not (*p* = 0.5).

### 3.4 Second primary malignancies

Second primary malignancies excluding non‐melanoma skin cancers were rare with only nine cases during follow‐up, resulting in a 3‐year cumulative incidence of 2.6% (Table S5).

### 3.5 Comparison with historical pre‐LM cohort

Between June 2015 and June 2019, 364 patients were treated with HDM‐ASCT. By comparing patients treated with HDM‐ASCT before (pre‐LM cohort) and after the approval of LM (LM cohort), we were able to investigate the impact of LM on PFS and OS. By chance, the chosen inclusion dates resulted in equal numbers of participants in the pre‐LM cohort and the LM cohort. Patients treated with HDM‐ASCT before June 2019 only rarely received LM outside of clinical trials and no other maintenance therapy was approved in Denmark at that time. This was confirmed by reviewing the electronic records of all 40 patients treated with HDM‐ASCT between January and June 2019. Of these, only three (7%) received LM, and in each case, LM was initiated just prior to approval while none received other types of maintenance.

Baseline characteristics for the LM and pre‐LM cohort were generally well balanced (Table 1). The most common induction regimen was VCD in the pre‐LM cohort and VRD in the LM cohort. The median time from diagnosis to HDM‐ASCT was slightly longer in the LM cohort (154 days vs. 146 days in the pre‐LM cohort), but constant throughout the years 2019–2022 (Table S6). The proportion of patients achieving VGPR or better after induction and HDM‐ASCT favored the LM cohort (84.8% vs. 78.7%). A higher prevalence of high‐risk cytogenetic alterations was seen in the LM cohort 25.0% versus 19.0%. In the LM cohort, cytogenetic results were missing in 76 (20.9%) of cases, whereas only 16 (4.4%) patients in the pre‐LM had missing cytogenetic data. Patients with missing cytogenetic data had similar clinical outcomes to patients with standard‐risk cytogenetics (Figure S4).

### 3.6 Progression‐free survival

**FIGURE 3:**
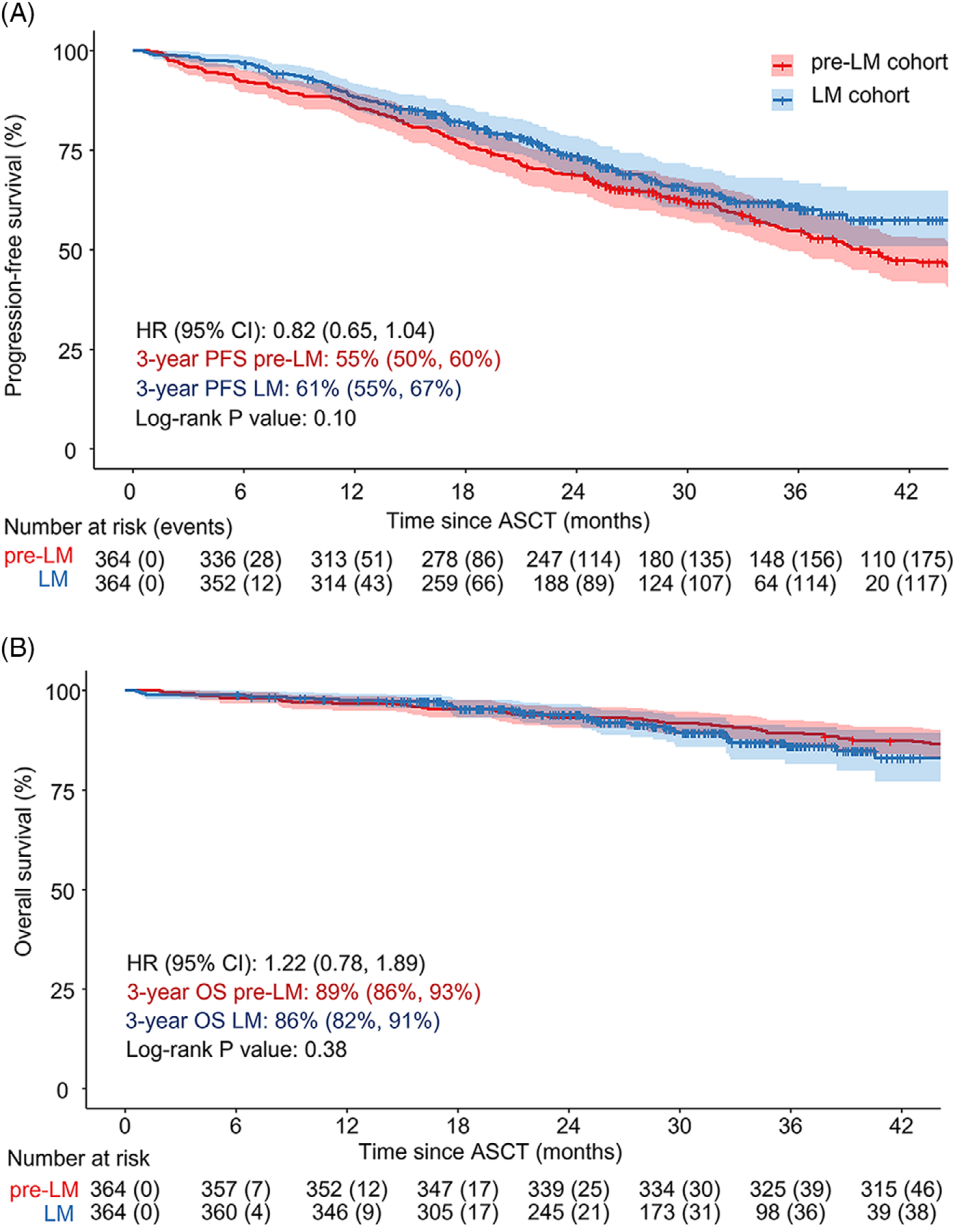
Progression‐free survival (PFS) (A) and overall survival (OS) (B) after high‐dose melphalan and autologous stem cell transplantation (HDM‐ASCT) for lenalidomide maintenance (LM) and pre‐LM cohort. Unadjusted Kaplan–Meier curves with time measured from date of autologous stem cell transplantation (ASCT). Unadjusted hazard ratios (HR) were calculated using cox proportional hazards regression. CI, confidence interval.

Median PFS from the time of HDM‐ASCT was not reached for the LM cohort. The 3‐year PFS rate was 61% (95% confidence interval [CI]: 55%, 67%) in the LM cohort versus 55% (95% CI: 50%, 60%) in the pre‐LM cohort; unadjusted HR 0.82 (0.65, 1.02) (Figure 3A). The risk of progression or death assessed by multivariate analysis adjusting for age, sex, cytogenetic risk, and ISS score was similar in the two cohorts: HR 0.84 (0.66, 1.07) (*p* = 0.16). In a subgroup analysis of patients with standard‐risk cytogenetics, we found a non‐significant trend toward longer PFS in the LM cohort; unadjusted HR 0.75 (0.55, 1.02) (*p* = 0.06) (Figure S2). In patients with high‐risk cytogenetics, no difference was observed between the LM and pre‐LM cohorts (Figure S3).

### 3.7 Overall survival

During follow‐up, 38 (10.4%) patients died in the LM cohort. Of these, 28 (70.2%) died in palliative care for treatment‐refractory MM. Five (13.2%) patients died because of infections, consisting of two (5.4%) cases of influenza A, two (5.4%) cases of bacterial sepsis, and one (2.7%) case of COVID‐19 (Table S7). The 3‐year OS was 86% (95% CI: 82%, 91%) in the LM cohort versus 89% (95% CI: 86%, 93%) in the pre‐LM cohort; unadjusted HR 1.22 (0.78, 1.89) (Figure 3B). The risk of death assessed by multivariate analysis adjusting for age, sex, cytogenetic risk, and ISS score was similar in the two cohorts: HR 1.16 (0.74, 1.82) (*p* = 0.5).

## 4 DISCUSSION

In this study, encompassing all Danish patients with MM treated with HDM‐ASCT since the introduction of LM, we found that LM was initiated in approximately three out of four patients. Approximately, one‐third of patients treated with LM discontinued this treatment within the first year, mainly due to toxicity. The most common cause of dose reduction was cytopenia, and the most common cause of toxicity leading to discontinuation was fatigue. In our landmark analysis, early discontinuation of LM was not associated with worse clinical outcomes. When comparing post‐transplantation PFS and OS in two cohorts of patients treated before and after the approval of LM, respectively, we found no differences.

In the randomized clinical trial, Myeloma XI, the leading cause of discontinuation of LM was progressive disease or death [3]. In contrast to this, we found that the leading cause of lenalidomide discontinuation was toxicity. One possible explanation to this may be that patients in a real‐world population including patients with co‐morbidity may be more prone to toxicity. Another explanation may be reduced adherence to a toxic treatment‐regimen outside of clinical trials. The most prevalent symptom leading to discontinuation in our study, fatigue, may have not led to discontinuation in a randomized clinical trial. This implies that certain toxicities, while seriously affecting patients’ quality of life, may receive less attention in clinical trials. We do, however, note that a large proportion of patients that discontinued LM due to toxicities were retreated with lenalidomide upon progression (eight out of 16), suggesting that the degree of toxicities that patients and physicians are willing to accept may be higher at the event of a relapse than during maintenance.

Randomized clinical trials with LM have so far only shown OS benefit in transplant eligible patients [1, 2, 3]. In contrast to most patients from the daily clinic, the selected subjects passing the eligibility criteria of such studies may be more fit and also more tolerant of extended antimyeloma therapy [18]. In this regard, it is positive that the 3‐year OS in our real‐world population, both in the LM (86.0%) and in pre‐LM cohort (89.0%), was comparable to that of transplant eligible patients who received LM in the Myeloma XI trial (87.5%). However, our results do not support any additional effect of LM to the efficacy of induction therapy and HDM‐ASCT.

Our results are markedly different than other previously published real‐world data, which found that LM was associated with PFS and OS benefits [9, 10, 11, 12]. However, one of the limitations of such studies is that they are liable to guarantee‐time bias, as the classifying event (initiation of LM) occurs after initiation of follow‐up. In some studies, the risk of guarantee‐time bias was mitigated by starting follow‐up 100 days post ASCT or excluding participants with progression or death during the first 100 days [9, 12]. Interestingly, we found similar PFS and OS for LM as seen in the Connect MM database (3‐year PFS, 61% vs. 56%, and 3‐year OS, 89% vs. 85%) [9]. However, our pre‐LM cohort had better 3‐year PFS (56% vs. 42%) and 3‐year OS (89% vs. 70%) compared to the no‐maintenance group of that study. In the Connect MM data, a large proportion of patients only received two‐drug induction regimens and at least 30% had not been exposed to lenalidomide prior to maintenance therapy. This could indicate that better induction regimens may make it more difficult to show benefits of LM. In this regard, it cannot be ruled out that our study could have shown a higher impact of LM with longer follow‐up.

A major strength of our study is the natural experiment resulting from a nationwide and simultaneous introduction of LM [19]. We take advantage of the randomness in being diagnosed and treated with HDM‐ASCT just before or just after June 2019, which effectively eliminates confounding by indication. This of course does not randomize for confounding effects introduced by the calendar year. In general, this tends to favor patients treated at later time points due to broader availability of the newest therapies from myeloma drug development [20]. However, the COVID‐19 pandemic may have preferentially harmed the LM cohort [21]. When assessing causes of death in this group of patients, we found that most patients died in palliative myeloma care and only one patient died of COVID‐19. Thus, we have not found direct evidence to support this hypothesis. Furthermore, the COVID‐19 pandemic did not seem to result in delayed HDM‐ASCT (Table S6). However, we cannot exclude that non‐deadly COVID‐19 infections in our LM cohort could have had a negative impact on PFS and OS, making a potential positive effect of LM less pronounced. Still, the case of COVID‐19 reducing PFS and OS in the LM cohort is not strong, as the results of our landmark analysis in patients (supposedly equally exposed to COVID‐19) with and without early discontinuation of LM did not support added benefit of continued LM. Another limitation is the shift in the preferred induction regimen from VCD in the pre‐LM cohort to VRD in the LM cohort. Although no randomized phase 3 trial has compared the two regimens directly, VRD is generally the preferred option and is believed to result in superior outcomes compared to VCD [5]. Furthermore, subgroup analysis from the CALGB 100104 study trended toward higher effect of LM in patients who had received lenalidomide induction [2]. In our interpretation, the difference in induction therapy between the pre‐LM and LM cohort is unlikely to explain the lack of difference in PFS and OS.

Our results are surprising, but we do caution interpretation. We show that the clinical outcomes of patients with MM in Denmark treated with HDM‐ASCT have not improved since the introduction of LM. Whether this observation is explained by residual confounding, or that improved induction and relapse treatment has diminished the effect of LM, cannot be established with certainty based on our results. With the emergence of quadruplet regimens and T‐cell redirecting therapies, which may yield even longer PFS and OS, it may be worthwhile to assess whether LM remains beneficial in future RCTs [22].

In conclusion, in this retrospective, nationwide, real‐world study, we found that LM was used in more than three out of four patients with MM after HDM‐ASCT. Approximately, one‐third of patients treated with LM discontinued this treatment within the first year, mainly due to toxicity. After a median follow‐up of 31 months from HDM‐ASCT, we did not observe PFS or OS benefits from the nationwide implementation of LM. Early discontinuation of LM due to toxicities did not impact PFS or OS. However, a difference in these outcomes could emerge with longer follow‐up.

## AUTHOR CONTRIBUTIONS

*Study concept and design*: Mads Harsløf and Agoston Gyula Szabo. *Design and testing of data instruments*: Agoston Gyula Szabo and Thomas Lund. *Data collection*: all authors. *Analysis of data*: Mads Harsløf and Agoston Gyula Szabo. *Interpretation of data*: all authors. *Drafting of manuscript*: Mads Harsløf. *Critical revision for important intellectual content*: all authors.

## CONFLICT OF INTEREST STATEMENT

All authors declare they have no conflicts of interest

## ETHICS STATEMENT

The study was approved by the Danish Data Protection Agency through the Region of Southern Denmark (Journal no. 18/22825) and the Danish Patient Safety Authority (Journal no. R‐22008541).

## PATIENT CONSENT STATEMENT

The ethics committee waived the requirement for informed consent.

## PERMISSION TO REPRODUCE MATERIAL FROM OTHER SOURCES

n/a

## CLINICAL TRIAL REGISTRATION (INCLUDING TRIAL NUMBER)

The authors have confirmed clinical trial registration is not needed for this submission.

## Supporting information

Supporting Information

## Data Availability

The datasets generated and analyzed during the current study are not publicly available due to the National and European Data Protection Regulation.
