# Supplementary material for: Nationwide implementation of lenalidomide maintenance in multiple myeloma: A retrospective, real‐world study
Source: EJHaem. 2024 Mar 27;5(2):316–24. doi: 10.1002/jha2.881 (PMC11020107; doi:10.1002/jha2.881)
Supplement: Supplementary file 1 — Supporting Information [file JHA2-5-316-s001.docx]

# Supplementary material

[Supplementary material 1](#_Toc144988854)

[Figure S1: Progression-free survival, landmark analysis 12 months after initiation of lenalidomide maintenance 3](#_Toc144988855)

[Figure S2: Progression-free survival (A) and Overall survival (B) in patients with standard-risk cytogenetics 4](#_Toc144988856)

[Figure S3: Progression-free survival (A) and Overall survival (B) in patients with high-risk cytogenetics 5](#_Toc144988857)

[Figure S4: Progression-free survival according to cytogenetic risk 6](#_Toc144988858)

[Table S1: Baseline characteristics of patients with or without discontinuation of lenalidomide maintenance within 6 months from initiation 7](#_Toc144988859)

[Table S2: Toxicities leading to dose reduction of lenalidomide maintenance 8](#_Toc144988860)

[Table S3: Toxicities leading to discontinuation of lenalidomide maintenance 9](#_Toc144988861)

[Table S4: Subsequent line of therapy in patients treated with lenalidomide maintenance and patients who did not receive lenalidomide maintenance 10](#_Toc144988862)

[Table S5: Second primary malignancy in the lenalidomide maintenance cohort 10](#_Toc144988863)

[Table S6: Time from diagnosis to HDM-ASCT 11](#_Toc144988864)

[Table S7: Causes of death in the lenalidomide maintenance cohort 11](#_Toc144988865)

## Figure S1: Progression-free survival, landmark analysis 12 months after initiation of lenalidomide maintenance

Kaplan-Meier curves for landmark analysis 12 months post initiation of lenalidomide maintenance. Unadjusted hazard ratios (HR) were calculated using cox proportional hazards regression. LM = lenalidomide maintenance.
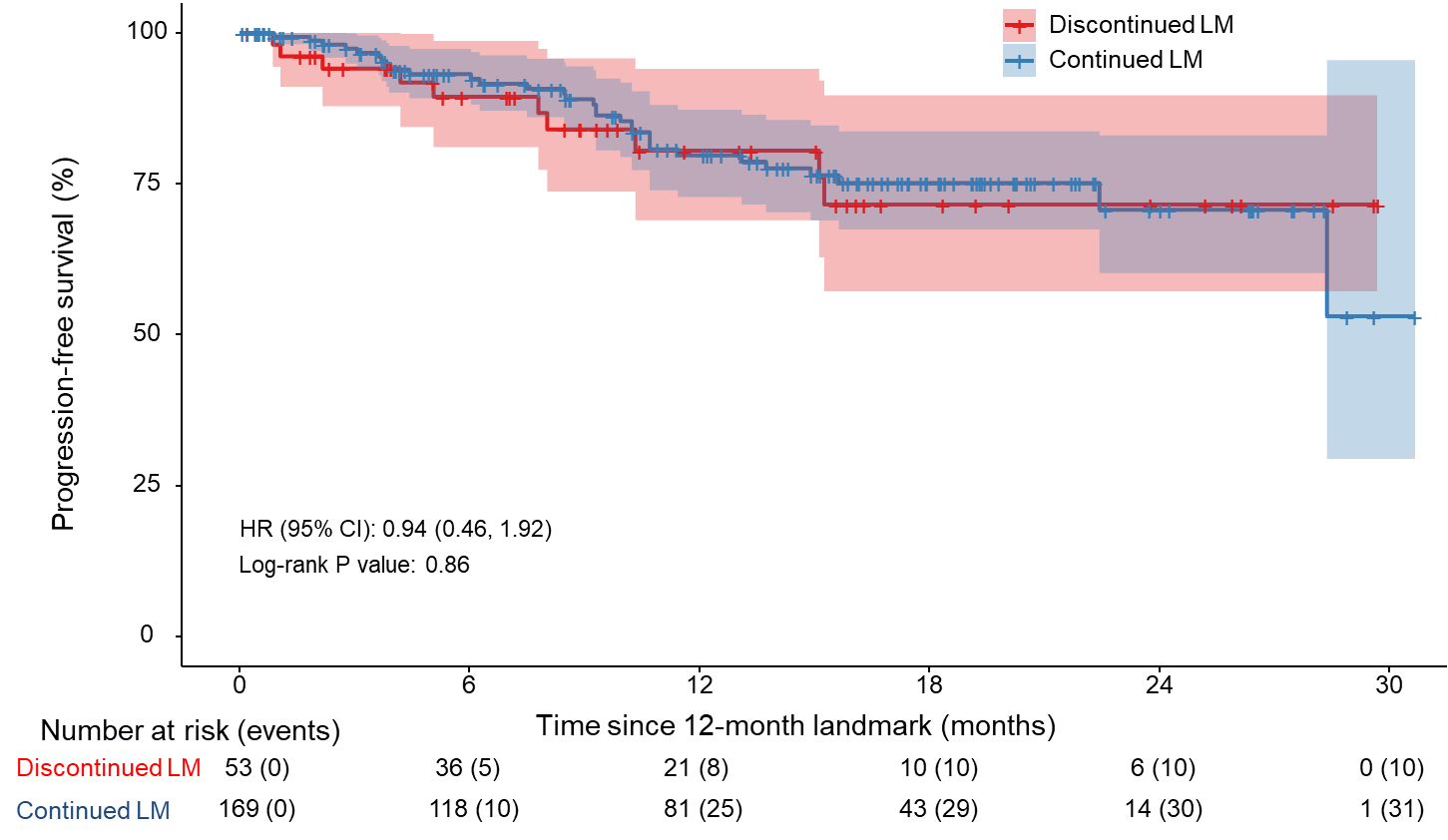


## Figure S2: Progression-free survival (A) and Overall survival (B) in patients with standard-risk cytogenetics


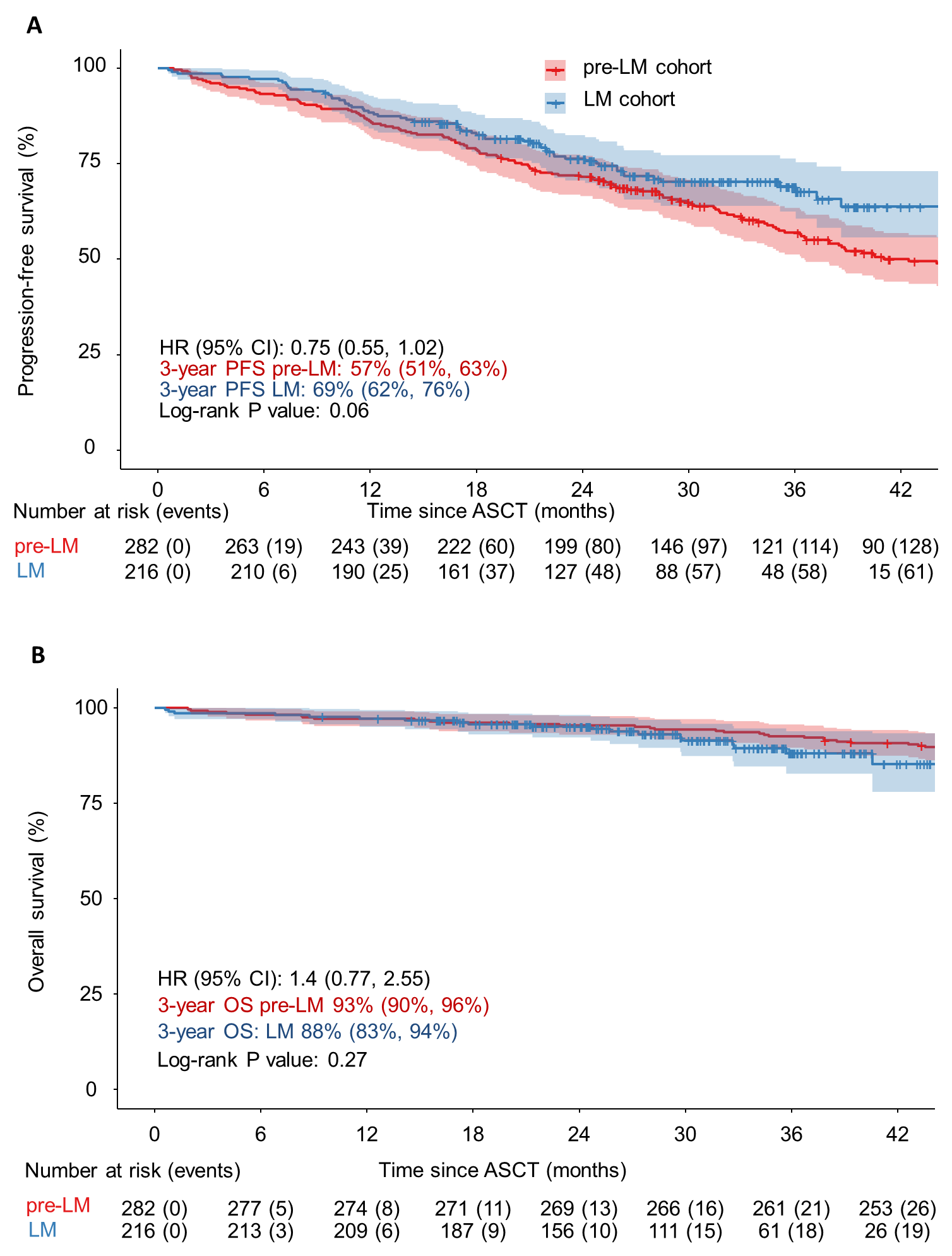
Unadjusted Kaplan-Meier curves with time measured from date of autologous stem cell transplantation (ASCT). Unadjusted hazard ratios (HR) were calculated using cox proportional hazards regression. LM = lenalidomide maintenance.

## Figure S3: Progression-free survival (A) and Overall survival (B) in patients with high-risk cytogenetics


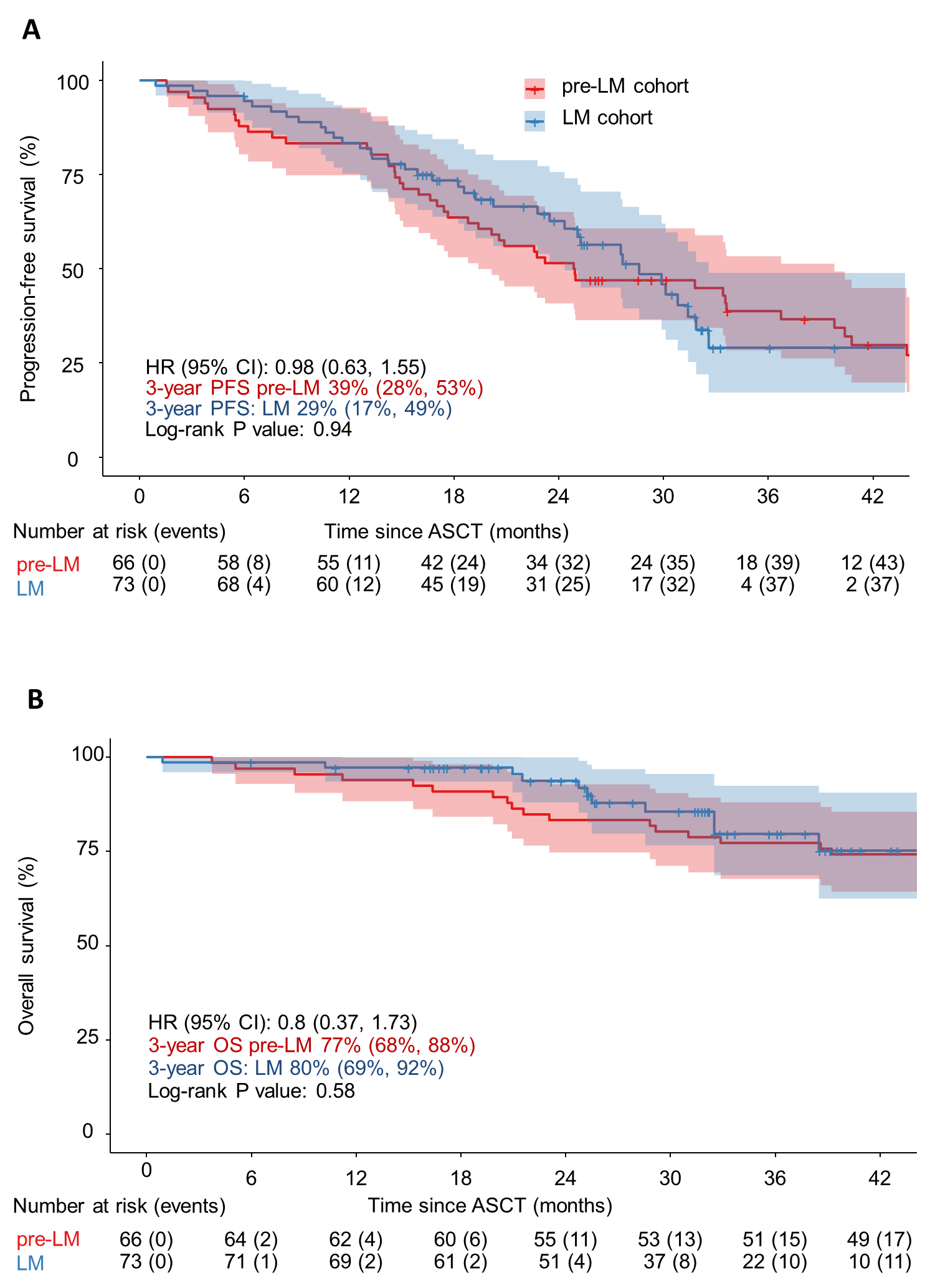
Unadjusted Kaplan-Meier curves with time measured from date of autologous stem cell transplantation (ASCT). Unadjusted hazard ratios (HR) were calculated using cox proportional hazards regression. LM = lenalidomide maintenance.

## Figure S4: Progression-free survival according to cytogenetic risk

Unadjusted Kaplan-Meier curves with time measured from date of autologous stem cell transplantation (ASCT). LM = lenalidomide maintenance.


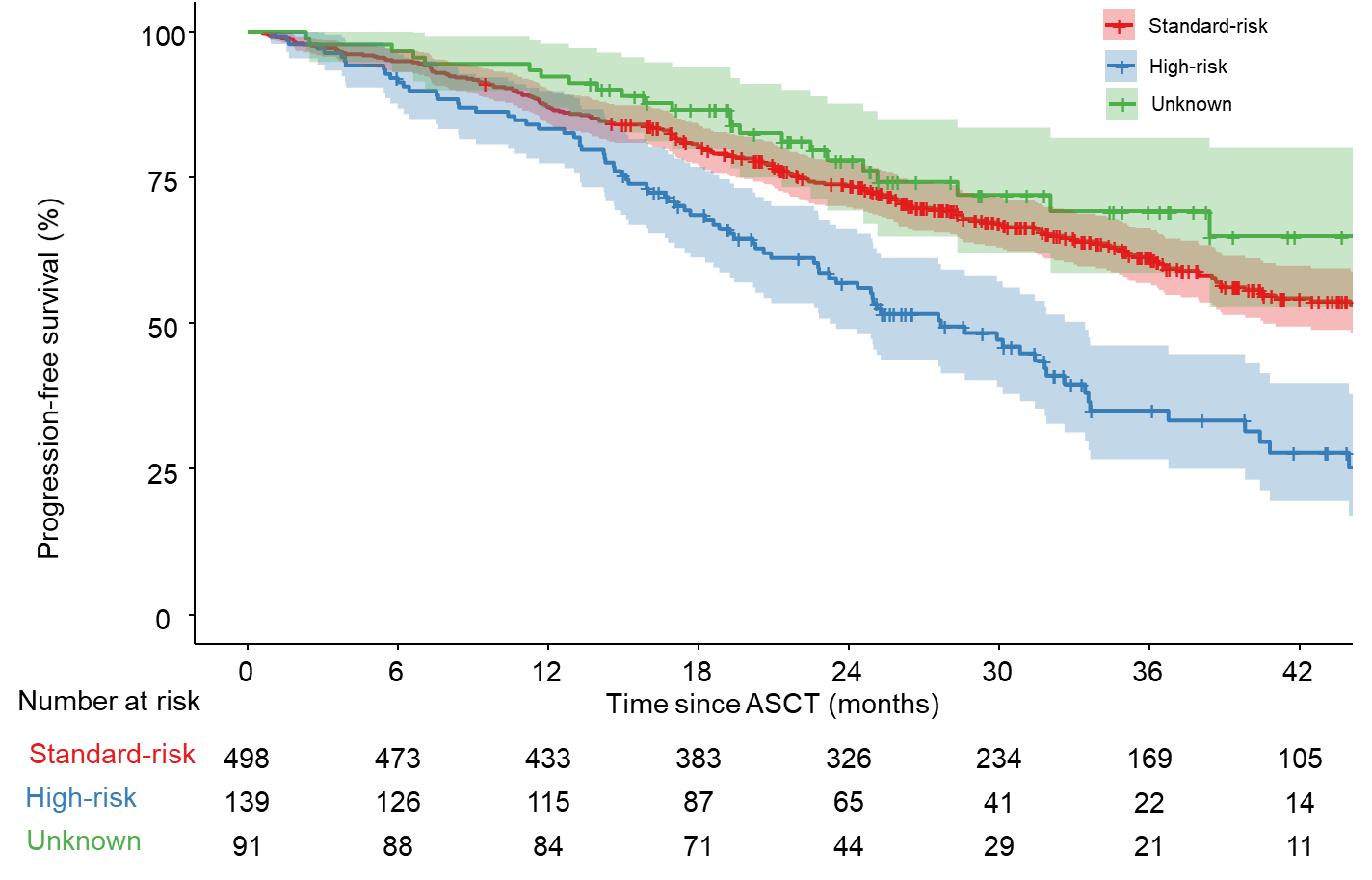


## Table S1: Baseline characteristics of patients with or without discontinuation of lenalidomide maintenance within 6 months from initiation

|  | Discontinued LM (N=51) | Continued LM (N=206) | Overall (N=257) |
| --- | --- | --- | --- |
| **Age** | 60.8 [56.0, 65.7] | 61.0 [55.8, 66.1] | 60.9 [55.9, 66.1] |
| **Sex** |  |  |  |
| Female | 22 (43.1%) | 88 (42.7%) | 110 (42.8%) |
| Male | 29 (56.9%) | 118 (57.3%) | 147 (57.2%) |
| **WHO performance status** |  |  |  |
| 0-1 | 41 (93.2%) | 159 (82.8%) | 200 (84.7%) |
| >1 | 3 (6.8%) | 33 (17.2%) | 36 (15.3%) |
| **Bone marrow infiltration (%)** | 45.5 [20.0, 60.0] | 50.0 [23.8, 70.0] | 50.0 [20.0, 70.0] |
| **Myeloma protein** |  |  |  |
| IgA | 8 (19.5%) | 36 (18.9%) | 44 (19.0%) |
| IgG | 23 (56.1%) | 115 (60.5%) | 138 (59.7%) |
| Light-chain | 6 (14.6%) | 32 (16.8%) | 38 (16.5%) |
| Other | 4 (9.8%) | 7 (3.7%) | 11 (4.8%) |
| **Calcium-ion > 1.35 (mmol/L)** | 11 (28.2%) | 33 (20.0%) | 44 (21.6%) |
| **Creatinine > 177 (µmol/L)** | 8 (18.2%) | 17 (8.9%) | 25 (10.6%) |
| **Hemoglobin < 6.2 (mmol/L)** | 10 (22.7%) | 60 (31.3%) | 70 (29.7%) |
| **Osteolytic lesions** | 29 (65.9%) | 148 (77.9%) | 177 (75.6%) |
| **Amyloidosis** | 1 (2.3%) | 2 (1.1%) | 3 (1.3%) |
| **Dialysis** | 1 (2.3%) | 3 (1.6%) | 4 (1.7%) |
| **Spinal cord compression** | 3 (6.8%) | 17 (8.9%) | 20 (8.5%) |
| **Beta-2-mikroglobulin (mg/L)** | 3.74 [2.97, 5.85] | 3.76 [2.40, 5.70] | 3.74 [2.50, 5.80] |
| **Albumin (g/L)** | 37.0 [33.5, 40.0] | 35.0 [30.0, 39.5] | 36.0 [30.0, 40.0] |
| **LDH > 205 (IU/L)** | 14 (32.6%) | 63 (33.5%) | 77 (33.3%) |
| **ISS** |  |  |  |
| I | 11 (28.2%) | 54 (29.0%) | 65 (28.9%) |
| II | 17 (43.6%) | 79 (42.5%) | 96 (42.7%) |
| III | 11 (28.2%) | 53 (28.5%) | 64 (28.4%) |
| **R-ISS** |  |  |  |
| I | 6 (18.2%) | 24 (14.7%) | 30 (15.3%) |
| II | 20 (60.6%) | 108 (66.3%) | 128 (65.3%) |
| III | 7 (21.2%) | 31 (19.0%) | 38 (19.4%) |
| **Cytogenetics available** | 37 (72.5%) | 162 (78.6%) | 199 (77.4%) |
| **Cytogenetic alteration** |  |  |  |
| t(4,14) | 5 (13.5%) | 22 (13.6%) | 27 (13.6%) |
| t(14,16) | 0 (0%) | 8 (4.9%) | 8 (4.0%) |
| del17p | 7 (18.9%) | 18 (11.1%) | 25 (12.6%) |
| High-risk cytogenetic* | 11 (29.7%) | 42 (25.9%) | 53 (26.6%) |
| **Induction regimen** |  |  |  |
| Other | 3 (5.9%) | 12 (5.8%) | 15 (5.8%) |
| VCd | 12 (23.5%) | 59 (28.6%) | 71 (27.6%) |
| VRd | 36 (70.6%) | 135 (65.5%) | 171 (66.5%) |
| **VGPR or better** | 46 (92.0%) | 178 (86.4%) | 224 (87.5%) |

For continuous variables median and IQR are reported and for categorical number of observations and percentage are shown. LDH=Lactate dehydrogenase, ISS=International Staging System, R-ISS=Revised-ISS. LM = lenalidomide maintenance. VCD= bortezomib- cyclophospamide-dexamethason. VRd = bortezomib-lenalidomide-dexamethason. VGPR = Very good partial response. *High-risk cytogenetic abnormalities were defined as t(4;14), t(14;16), and del(17p).

## Table S2: Toxicities leading to dose reduction of lenalidomide maintenance

Number and percentages of patients experiencing a given toxicity requiring dose reduction. One patient may have had several toxicities leading to dose reduction.

| Toxicity | n (%) |
| --- | --- |
| Cytopenia | 51 (49.0%) |
| Fatigue | 24 (23.1%) |
| Rash | 13 (12.5%) |
| Neuropathy | 9 (8.7%) |
| Diarrhea | 6 (5.8%) |
| Other | 7 (6.7%) |
| Infections | 6 (5.8%) |
| Other gastrointestinal | 5 (4.8%) |
| Cramps | 5 (4.8%) |
| Musculoskeletal | 3 (2.9%) |
| Thrombotic events | 1 (1.0%) |
| Other skin reaction | 1 (1.0%) |

## Table S3: Toxicities leading to discontinuation of lenalidomide maintenance

Number and percentages of patients experiencing a given toxicity requiring discontinuation of lenalidomide maintenance. One patient may have had several toxicities leading to discontinuation.

| Toxicity | n (%) |
| --- | --- |
| Fatigue | 23 (30.7%) |
| Cytopenia | 19 (25.3%) |
| Neuropathy | 13 (17.3%) |
| Rash | 8 (10.7%) |
| Diarrhea | 7 (9.3%) |
| Other | 7 (9.3%) |
| Other gastrointestinal | 5 (6.7%) |
| Musculoskeletal | 4 (5.3%) |
| Infections | 4 (5.3%) |
| Second primary malignancy | 4 (5.3%) |
| Thrombotic events | 3 (4.0%) |
| Cramps | 2 (2.7%) |
| Other neurological complication | 2 (2.7%) |
| Liver | 1 (1.3%) |
| Other skin reaction | 1 (1.3%) |

## Table S4: Subsequent line of therapy in patients treated with lenalidomide maintenance and patients who did not receive lenalidomide maintenance

| **Subsequent line of therapy** | | | Lenalidomide (N=76) | | No lenalidomide maintenance (N=32) | | Overall (N=108) |
| --- | --- | --- | --- | --- | --- | --- | --- |
| **Regimens** | | |  | |  | |  |
| daratumumab-lenalidomide-dexamethason | | | 20 (26.3%) | | 16 (50.0%) | | 36 (33.3%) |
| daratumumab-bortezomib-dexamethason | | | 17 (22.4%) | | 2 (6.3%) | | 19 (17.6%) |
| carfilzomib-lenalidomide-dexamethason | | | 8 (10.5%) | | 3 (9.4%) | | 11 (10.2%) |
| daratumumab-pomalidomide-dexamethason | | | 9 (11.8%) | | 1 (3.1%) | | 10 (9.3%) |
| bortezomib-lenalidomide-dexamethason | | | 3 (3.9%) | | 1 (3.1%) | | 4 (3.7%) |
| carfilzomib-cyclophospamide-dexamethason | | | 3 (3.9%) | | 1 (3.1%) | | 4 (3.7%) |
| Other | | | 16 (21.1%) | | 8 (25.0%) | | 24 (22.2%) |
| **Drug-exposure** | | |  | |  | |  |
| daratumumab | | | 54 (71.1%) | | 22 (68.8%) | | 76 (70.4%) |
| lenalidomide | | | 33 (43.4%) | | 23 (71.9%) | | 56 (51.9%) |
| bortezomib | | | 22 (28.9%) | | 4 (12.5%) | | 26 (24.1%) |
| carfilzomib | | | 17 (22.4%) | | 8 (25.0%) | | 25 (23.1%) |
| pomalidomide | | | 12 (15.8%) | | 2 (6.3%) | | 14 (13.0%) |
| cyclophosphamide | | | 5 (6.6%) | | 1 (3.1%) | | 6 (5.6%) |
|  | | |  | |  | |  |
|  |  |  | |  | |  |  |

## Table S5: Second primary malignancy in the lenalidomide maintenance cohort

| Second primary malignancy | n |
| --- | --- |
| Breast cancer | 2 |
| Bladder cancer | 1 |
| Gastro-intestinal cancer | 1 |
| Lung cancer | 1 |
| Sarcoma | 1 |
| Ovarian cancer | 1 |
| Malignant melanoma | 1 |
| Myelodysplastic neoplasm | 1 |
|  |  |

## Table S6: Time from diagnosis to HDM-ASCT

| Year of HDM-ASCT | median (IQR) |
| --- | --- |
| 2015 | 142 days (130, 168) |
| 2016 | 137 days (127, 156) |
| 2017 | 153 days (136, 171) |
| 2018 | 148 days (135, 169) |
| 2019 | 155 days (137, 180) |
| 2020 | 153 days (139, 178) |
| 2021 | 154 days (139, 182) |
| 2022 | 155 days (142, 187) |

## Table S7: Causes of death in the lenalidomide maintenance cohort

| Cause of death | n (%) |
| --- | --- |
| Myeloma in palliative care | 28 (73.7) |
| Infections |  |
| - Sepsis | 2 (5.3) |
| - Influenza A | 2 (5.3) |
| - Covid-19 | 1 (2.6) |
| Vascular disease | 2 (5.3) |
| Other malignancy | 1 (2.6) |
| Suicide | 1 (2.6) |
| Cardiac disorder | 1 (2.6) |
